# The comparisons of non‐linear models to describe the growth performance of Lori‐Bakhtiari sheep

**DOI:** 10.1002/vms3.1527

**Published:** 2024-07-23

**Authors:** Saheb Foroutanifar, Majid Khaldari

**Affiliations:** ^1^ Department of Animal Science College of Agriculture and Natural Resources Razi University Kermanshah Iran; ^2^ Department of Animal Science Faculty of Agriculture Lorestan University Khorramabad Iran

**Keywords:** body weights, growth curves, non‐linear functions, sheep

## Abstract

**Background:**

The study of growth traits is of interest to many animal scientists, regardless of specialization, due to the economic importance of growth rate, mature weight and other related traits.

**Objective:**

This study aimed to compare six non‐linear models for describing the growth of Lori‐Bakhtiari sheep.

**Methods:**

In order to collect weight data, 85 lambs (41 males and 44 females) were reared from birth to 140 days of age, and their growth patterns were recorded by measuring their body weight at 10‐day intervals. Various mathematical functions, including the negative exponential, Brody, Gompertz, Logistic, Morgan–Mercer–Flodin (MMF) and Weibull, were used to model the relationship between body weight records and age.

**Results:**

The results showed that the MMF and Gompertz models provided the best fit to the body weight data, whereas the negative exponential model exhibited the worst fit. In all models, the asymptotic weight of male lambs was higher than females. The research also revealed differences in growth patterns between male and female lambs. Overall, females had a lower absolute growth rate than males, but they reached their peak growth at an earlier period, and their growth rate declined faster.

**Conclusions:**

The differences in growth patterns between males and females indicate the importance of analysing male and female data separately when describing growth. As a result, Gompertz model can be recommended to Lori‐Bakhtiari female and male lamb breeders to determine more accurate growth traits. In addition, it should be considered that feeding male and female lambs separately according to absolute growth rate values may increase growth performance.

## Graphical abstract

This study aimed to compare six non‐linear models for describing the growth of Lori‐Bakhtiari sheep. The MMF and Gompertz models provided the best fit. The research also revealed differences in growth patterns between male and female sheep. Females had a lower absolute growth rate compared to males, and they reached their peak earlier and experienced a faster decline in AGR.

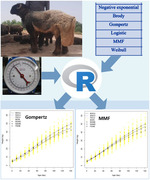

## 1 INTRODUCTION

Over 25 sheep breeds have adapted to diverse climatic conditions in Iran, and nearly all of them are reared mainly for meat production (Valizadeh, 2010; Vatankhah et al., 2004). Approximately 35% of the total red meat production in Iran is provided by sheep breeding (ICTC, 2022). Although Lori‐Bakhtiari sheep exhibit low reproductive performance, they are the most heavily weighting breed in Iran with a large fat tail that reaches the hocks (Vatankhah et al., 2004).

Growth is an important trait in farm animals and is measured as an increase in live weight (de Fatima Sieklicki et al., 2016). However, it can be challenging to explain extensive weight data on animals unless they are condensed into a few key parameters. To address this issue, growth curve parameters are utilized to encapsulate the information into manageable statistics (Makgopa et al., 2023).

The study of growth traits is of interest to many animal scientists, regardless of specialization, because of the economic importance of growth rate, mature weight and other related attributes (Makgopa et al., 2023). For animal breeders, understanding the genotypic and phenotypic relationships among weight data, time to maturity and growth rate across all stages of growth is a critical component in the design of breeding programs (Smith et al., 1976). Animal breeders can also refer to the biological implications of growth curve parameters and their associations with other traits as guidance in developing a breeding program intended to modify the growth curves of animals (Bilgin, Emsen et al., 2004). Additionally, growth curves are essential for determining the best slaughtering age and feeding system for animals and identifying the effects of selection on the basis of curve parameters for weight at a definite age (Blasco & Gómez, 2010).

Different non‐linear growth curve equations have been used to describe the relationship between the body weight and age of sheep (Lambe et al., 2006). Therefore, the present research compared six alternative non‐linear models for describing the growth of Lori‐Bakhtiari lambs from birth to 5 months of age.

## 2 MATERIALS AND METHODS

This study was conducted at Lorestan province, which is located in the western part of Iran, 33.3° to the north, 48.18° to the east and 1125 m above sea level. The flock of sheep used in this study was obtained from a sheep and goat breeding institute in Gahare Dorood. The flock was allowed to graze over ranges and pastures in spring and summer and fed intensive condition in autumn and winter. Ewes typically lambing three times in every 2 years. If lambing occurs in winter, the lambs graze on ranges in spring with their mothers; otherwise, they are fed intensively. The lambs were weaned at approximately 80 days of age, and each lamb was fed an average of 300 g of concentrate feed (which contains 11.95% crude protein, 2.74 Mcal/kg metabolic energy, 0.54% calcium and 0.3% phosphorus). After weaning, they are fed up to 5 months of age and then sold after selection.

This study involved 85 lambs (41 males and 44 females), whose growth data consisted of body weights (kilograms) recorded at 10‐day intervals from birth to 140 days of age. The negative exponential, Brody, Gompertz, Logistic, Morgan–Mercer–Flodin (MMF) and Weibull non‐linear equations were used to describe the relationship between weight and age from birth to 140 days of age (Table 1). The biological interpretation of the parameters in the aforementioned functions is as follows: W(*t*) is the body weight of an animal at time *t*, α denotes the asymptotic limit of weight when *t* approaches infinity, β is a scale parameter related to birthweight, k represents the relative growth rate at which asymptotic weight is reached, m is the shape parameter defining the inflection point of a curve, and e represents the random error. To describe the relationship between body weight and age, each of the six non‐linear functions was fitted separately to the body weight data of the males, females and all lambs. Model fitting was performed using R statistical computing and graphics software (v3.3.2). Non‐linear least squares regression using the *nls ()* function in the R *stats* (v3.3.2) package (R Core Team, 2016) was carried out to estimate parameters on the basis of the different functions.

**TABLE 1:** The non‐linear functions used to describe the growth curve of Lori‐Bakhtiari lambs.

Model	Equation	Number of parameters	Reference
Negative exponential	W(t)=α[1−exp(−kt)]+e	2	Brown et al. (1976)
Brody	W(t)=α[1−βexp(−kt)]+e	3	Brody (1945)
Gompertz	W(t)=αexp[−βexp(−kt)]+e	3	Laird et al. (1965)
Logistic	W(t)=α/[1+βexp(−kt)]+e	3	Nelder (1961)
MMF	W(t)=α−[(α−β)/(*1+* ${\left(kt\right)}^{m}$)]+e	4	Morgan et al. (1975)
Weibull	$W\left(t\right)=\alpha -\beta \;\exp\left(-kt^{m}\right)+e$	4	Fekedulegn et al. (1999)

The goodness of fit of each model was determined using the following criteria: log likelihood; Akaike information criterion AIC = *n* × ln (SSE/*n*) + 2*p*; Bayesian information criterion BIC = *n* × ln (SSE/*n*) + *p* × ln(*n*); root mean square error RMSE = √[(1/*n*) × SSE)] and adjusted coefficient of determination *R* ^2^ = 1–((*n*–1)/(*n*–*p*)) (1–(1–(SSE/SST))). In these equations, SSE is the error sum or squares, SST denotes the total sum of squares, *n* represents the number of observations, and *p* refers to the number of parameters. After identifying the model with the best fit, the absolute growth rate (AGR) was estimated based on the first derivative of this function with respect to time (d*W*/d*t* where *W* and *t* are the best fitted model function and time, respectively); this estimate graphically shows the daily growth rate.

## 3 RESULTS

### 3.1 Model parameters

The least square means estimates and standard errors of *α*, *β*, *k* and *m* for each growth model are shown in Table 2. The estimates of these parameters varied among the models. For example, the greatest value for parameter *α* was obtained by the Brody model, with values of 268.92, 99.81 and 218.03 for males, females and all lambs, respectively. This parameter is used to estimate the asymptotic weight, but it can be interpreted as representative of mature weight. The second highest values were estimated using the MMF model (113.51, 78.82 and 124.04 for males, females and all lambs, respectively), whereas Logistic model produced lowest estimates (53.71, 42.86 and 47.67 for males, females and all lambs, respectively). In all models, the *α* of male lambs was higher than that of female lambs. The highest *k* values were estimated by the Logistic model (0.03), whereas the lowest values were obtained by the Brody model (0.0016, 0.003714 and 0.0014 for all lambs, females and males, respectively). The highest *β* values were estimated by the Weibull function (81.12, 72.02 and 51.71 for all lambs, males and females, respectively), whereas the lowest were obtained with the Brody model (0.99, 0.98 and 0.96, respectively). The Weibull function estimated slightly lower *m* values than the MMF model, but both models gave higher values for male lambs than for female lambs.

**TABLE 2:** Mean growth curve parameters (±standard error) of six growth functions in Lori–Bakhtiari lambs.

Growth model	α	β	k	m
Negative exponential				
Both	94.78 ± 5.57	–	0.0046 ± 4e − 04	–
Male	91.92 ± 5.44	–	0.0055 ± 5e − 04	–
Female	59.63 ± 2.46	–	0.0085 ± 6e − 04	–
Brody				
Both	218.03 ± 63.42	0.99 ± 0.01	0.0016 ± 5e − 04	–
Male	268.92 ± 106.89	0.98 ± 0.01	0.0014 ± 6e − 04	–
Female	99.81 ± 14.35	0.96 ± 0.01	0.0037 ± 7e − 04	‐
Gompertz				
Both	56.67 ± 1.68	2.52 ± 0.04	0.0168 ± 7e − 04	–
Male	64.47 ± 2.32	2.39 ± 0.04	0.0156 ± 8e − 04	–
Female	49.28 ± 1.53	2.21 ± 0.04	0.0181 ± 9e − 04	–
Logistic				
Both	47.67 ± 0.84	7.45 ± 0.24	0.0313 ± 9e − 04	–
Male	53.71 ± 1.11	6.74 ± 0.23	0.0294 ± 0.001	–
Female	42.86 ± 0.83	5.81 ± 0.22	0.032 ± 0.0011	–
MMF				
Both	124.04 ± 27.83	5.19 ± 0.42	0.0043 ± 0.0012	1.24 ± 0.08
Male	113.51 ± 22.55	5.66 ± 0.54	0.0054 ± 0.0015	1.35 ± 0.11
Female	78.82 ± 12.17	5.08 ± 0.48	0.0048 ± 0.0016	1.33 ± 0.11
Weibull				
Both	84.9 ± 16.72	81.12 ± 16.92	0.0022 ± 3e − 04	1.18 ± 0.07
Male	77.77 ± 11.71	72.02 ± 11.96	0.0017 ± 4e − 04	1.28 ± 0.09
Female	56.86 ± 6.31	51.71 ± 6.52	0.0027 ± 6e − 04	1.24 ± 0.08

*Note*: *α*, *β*, *k* and *m* are estimated growth parameters.

### 3.2 Goodness of fit

The goodness‐of‐fit measures (adjusted *R* ^2^, log likelihood, RMSE, AIC and BIC) are presented in Table 3. The model with the highest adjusted *R* ^2^ (*R* ^2^_adj_) and log likelihood values and the lowest RMSE, AIC and BIC values was accepted as the best model. Adjusted coefficient of determinations (*R* ^2^_adj_) was high for all models, and ranging from 0.89 to 0.93, suggesting that the models adequately described the collected data. The Gompertz model was found to be the most effective for both male and female lambs, according to the comparison based on the log likelihood, RMSE, AIC and BIC. For these criteria, however, the MMF model and Weibull models’ values were somewhat close to those of the Gompertz model. For all lambs, the MMF model performed better than any other models. Nevertheless, the lower AIC and BIC values of Gompertz model relative to those of the MMF model indicated that the extra *m* parameter in the MMF model did not effectively improve the fit for data on male and female lambs.

**TABLE 3:** The goodness of fit of six implemented growth models in Lori‐Bakhtiari lambs.

Growth model	Log likelihood	AIC	BIC	RMSE	*R* ^2^
Negative exponential					
Both	−3144.49	6294.98	6309.99	4.22	0.89
Male	−1559.08	3124.15	3137.09	4.08	0.91
Female	−1484.26	2974.53	2987.45	3.63	0.90
Brody					
Both	−3097.45	6202.90	6222.91	4.04	0.90
Male	−1500.82	3009.64	3026.9	3.67	0.93
Female	−1422.41	2852.83	2870.05	3.24	0.92
Gompertz					
Both	−3099.94	6207.89	6227.9	4.05	0.90
Male	−1496.12	3000.24	3017.5	3.64	0.93
Female	−1418.97	2845.93	2863.16	3.22	0.92
Logistic					
Both	−3121.28	6250.56	6270.57	4.13	0.90
Male	−1504.33	3016.66	3033.91	3.69	0.93
Female	−1427.62	2863.25	2880.47	3.27	0.92
MMF					
Both	−3038.53	6087.06	6112.08	3.83	0.91
Male	−1496.24	3002.48	3024.05	3.64	0.93
Female	−1418.67	2847.34	2868.87	3.22	0.92
Weibull					
Both	−3094.54	6199.08	6224.1	4.03	0.9
Male	−1496.09	3002.18	3023.75	3.64	0.93
Female	−1418.51	2847.03	2868.56	3.22	0.92

### 3.3 Predicted weight

**FIGURE 1:**
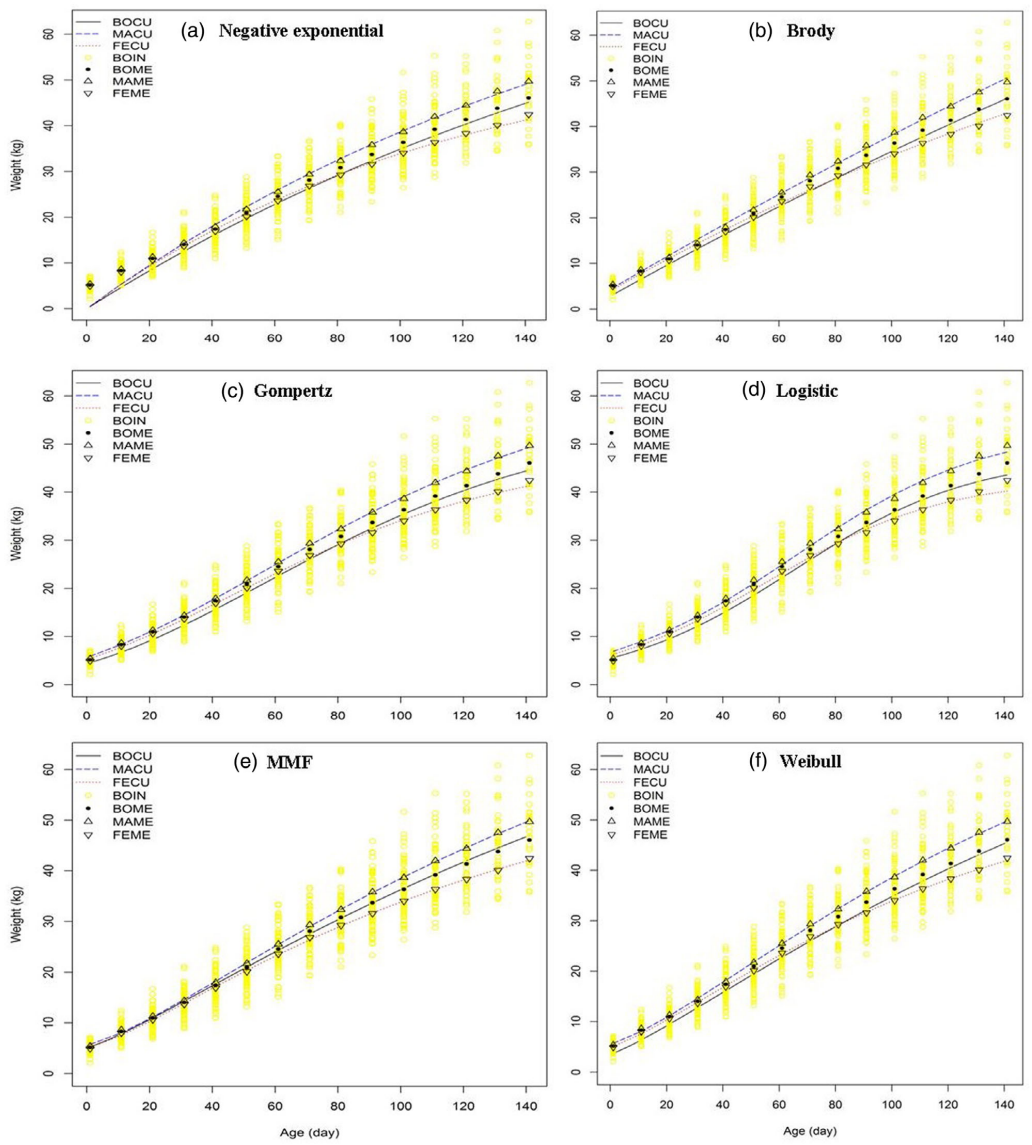
Fitted growth curves of Lori‐Bakhtiari sheep breed predicted by the negative exponential (a), Brody (b), Gompertz (c), Logistic (d), Morgan–Mercer–Flodin (e) and Weibull (f) models. BOCU, MACU and FECU are the predicted values for all lambs, males and females, respectively; BOIN is the observed weight for each lamb; BOME, MAME and FEME are the mean of weight data in each day for all lambs, males and females, respectively.

Figure 1 illustrates the weight values predicted by models based on the average values presented in Table 3 and the observed data from birth to 140 days. Overall, the negative exponential (Figure 1a) and Brody (Figure 1b) models exhibited an exponential pattern, whereas the Gompertz (Figure 1c), Logistic (Figure 1d), MMF (Figure 1e) and Weibull (Figure 1f) models showed a sigmoid pattern. The body weight of animals increased as they grew older. Females had a lower weight at birth, and the weight difference between male and female lambs continued to increase as they grew up to day 140. When all lambs were used for model fitting, all models, except for the MMF, underestimated body weight at all ages. When all lambs were fitted to different models, it became evident that all models except the MMF underestimated body weight across all ages. This finding indicates that only the MMF function was able to appropriately describe the growth curve using data from all lambs. Conversely, when male and female lambs were considered separately, all of the curves, except for the negative exponential, appropriately fit the growth data. Nonetheless, small deviations could be found in the Gompertz model.

### 3.4 Absolute growth rate (AGR)

**FIGURE 2:**
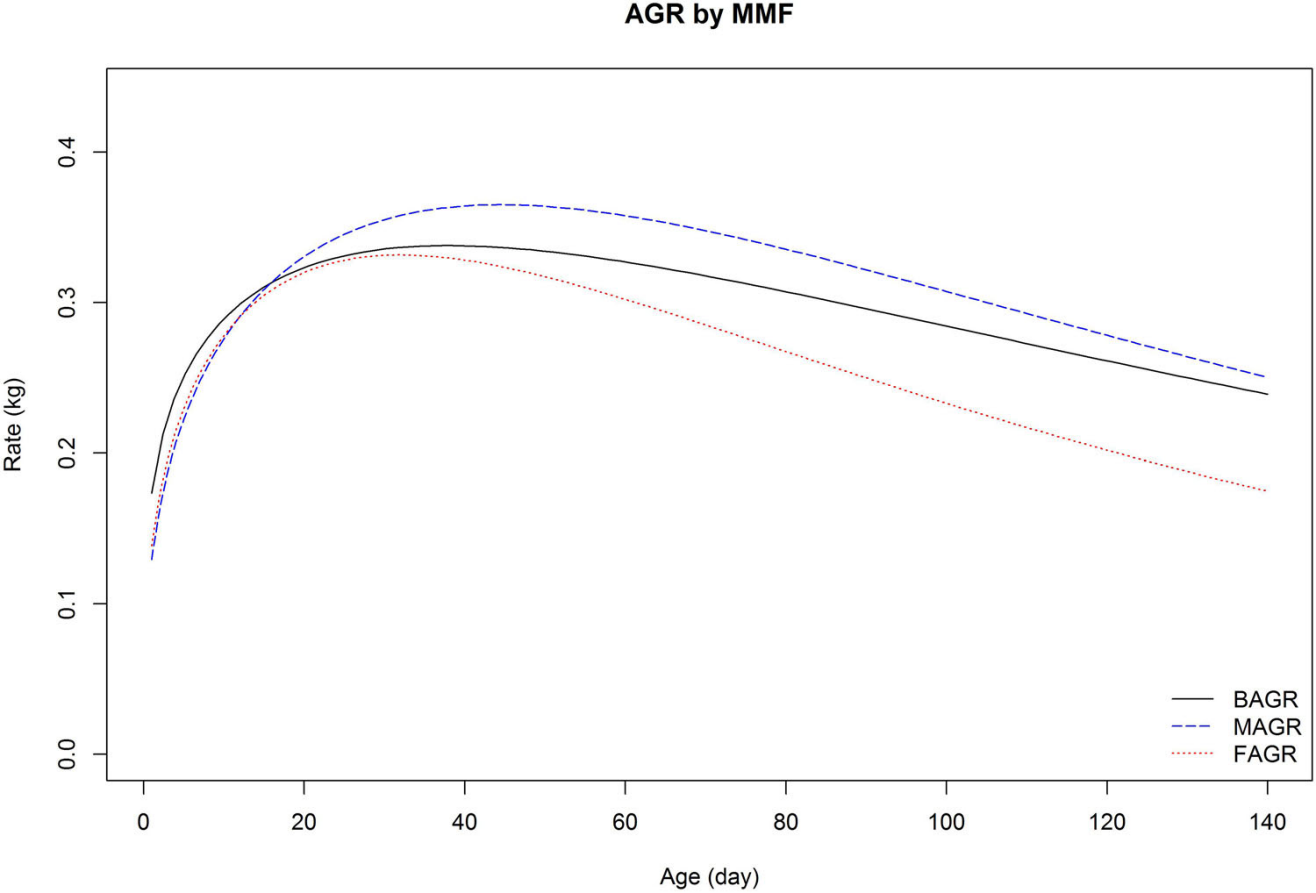
Absolute growth rate (AGR) described by Morgan–Mercer–Flodin (MMF) function to females (FAGR) and males (MAGR) and all Lori‐Bakhtiari lambs (BAGR).

The AGR, derived based on the first derivative of the MMF model with respect to time, is presented in Figure 2. On this graph, the horizontal axis (*X* axis) represents age in days, whereas the vertical axis (*Y* axis) shows AGR in kilograms per day (kg/day). The AGR values peaked quickly and then gradually decreased over time. The maximum values at the peak were 0.36, 0.33 and 0.34 kg/day were obtained at days 44, 32 and 38 for males, females and all lambs, respectively. These results show that the maximum growth rate occurred early in both sexes, with females reaching this peak sooner. The AGR declined more rapidly in females compared to males, reaching 0.17 and 0.25 kg/day at day 140 for females and males, respectively. The AGR provides valuable information for assessing animal development efficiency and determining the optimal slaughter age.

## 4 DISCUSSION

This study is the first to compare six different non‐linear models for describing the growth patterns of Lori‐Bakhtiari sheep. Although the results showed that all models provided adequate descriptions of growth curves, there were considerable differences in the estimates of *α*, *β*, *k* and *m*. Other studies have also reported variation in *α*, *β* and *k* values obtained by different non‐linear growth models when analysing weight–age data for different breeds of the same species. These findings suggest that estimation of parameters is affected by the specific model used, the number of parameters in the used models, birth type, interval of test day records used, the age taken last body weight record and animal species and breed under study (Bahreini Behzadi et al., 2014; Brown et al., 1976; Ghavi Hossein‐Zadeh & Golshani, 2016; Goliomytis et al., 2006; Güler et al., 2023).

The *α* values of male lambs were higher than those of female lambs in all models, reflecting that males reached a greater mature weight than females. Previous studies consistently demonstrate that male lambs outperform female lambs in terms of growth rates and mature weights, irrespective of factors such as breed and growth model (Bangar et al., 2021; Sharif et al., 2021). The Logistic model underestimated the mature weights of male and female Lori‐Bakhtiari sheep. Similarly, Ghavi Hossein‐Zadeh (2015) and Bahreini Behzadi et al. (2014) ranked the Brody function ahead of the Gompertz and Logistic models in terms of asymptotic body weight of Iranian Shall and Baluchi fat‐tail sheep, respectively. However, these authors obtained a lower *α* when using the Brody and other models. Other researchers likewise derived the smallest *α* values with the Logistic model (Bahreini Behzadi et al., 2014; Gbangboche et al., 2008; Ghavi Hossein‐Zadeh, 2015; Kopuzlu et al., 2013).

The parameter *k* represents the maturation rate, and it helps to describe the shape of the curve. It becomes a uniquely functioning parameter when it is used to relate size to productivity (Kopuzlu et al., 2013). Although *k* represents the maturity index, it does not give the same value across different models. In this study, the models like Brody model, which generated the highest *α* (mature weight), also produced the lowest *k* (relative growth rate); in contrast, the Logistic model obtained the lowest *α* and the highest *k*. This negative relationship between *α* and *k* indicates that animals with a high mature weight need more time to reach this weight. The relative growth rate of females was higher than that of males; thus, females reached maturity earlier than males. The Brody model obtained a higher *α* and lower *k* for males and females compared to other Iranian fat‐tail sheep breeds (Bahreini Behzadi et al., 2014; Bathaei & Leroy, 1996; Ghavi Hossein‐Zadeh & Golshani, 2016; Hojjati & Ghavi Hossein‐Zadeh, 2017).

Parameter *β* denotes the scale parameter related to birthweight. The Brody, Logistic and Gompertz models were utilized in the studies of Iranian fat‐tail sheep breeds including Shall, Mehraban and Baluchi. The findings were consistent with those of the present research in terms of *β* (Bahreini Behzadi et al., 2014; Ghavi Hossein‐Zadeh, 2015; Hojjati & Ghavi Hossein‐Zadeh, 2017).

Some growth curve functions such as Logistic, Gompertz and Von Bertalanffy have a fixed point of inflections unlike the models have parameter m which indicates flexible inflection point such as Richard, MMF and Weibull (Bilgin, Esenbuga et al., 2004). In models which contain *m* in their functions, the boundaries of this parameter are simply a biological interpretation. The MMF and Weibull models yielded a larger *m* for male lambs than for females, indicating that males reached inflection at an earlier stage than females. Similarly, several studies using non‐linear growth models containing *m* parameters reported a positive *m*, whereas others found a negative value (Brown et al., 1976; da Silva et al., 2012; Ghavi Hossein‐Zadeh, 2015; Kopuzlu et al., 2013; Tariq et al., 2013). It can be said that the reason why the *m* parameter varies in studies and has positive‐negative signs depends on the growth curve models used in the study, the care and feeding conditions and the breed of the sheep. Given the negative correlation between *β* and *m*, the values of the latter vary because of the values of *β* (Brown et al., 1976).

On the basis of the different evaluation measures, the MMF and Gompertz models exhibited the best fit to the body weight data, whereas the negative exponential model had the worst fit. These results are confirmed by Figure 1. Previous studies on sheep have identified different functions as the most appropriate models for representing body weight. As was the case in the current study, the Gompertz function was selected as a suitable model for Texel and Scottish Blackface lambs (Lambe et al., 2006), Morkaraman lambs (Topal et al., 2004). Tariq et al. (2013) determined that the MMF model provided the best fit to body weight data of all male and female Mengali sheep. The Logistic model has been demonstrated to have the best goodness of fit for Moghani (Ghavi Hossein‐Zadeh, 2017), Santa Ines (da Silva et al., 2012), Norduz (Daskiran et al., 2010) and Awasi (Tekel et al., 2005) sheep breeds. The Brody model was selected as the most accurate representation of data for Hemsin (Kopuzlu et al., 2013), Baluchi (Bahreini Behzadi et al., 2014) and Mehraban (Hojjati & Ghavi Hossein‐Zadeh, 2017) lambs. These variations can be attributed to differences in the genetic background of breeds and environmental factors affecting the growth of lambs.

## 5 CONCLUSION

Six growth functions were compared to ascertain their suitability for describing growth data of Lori‐Bakhtiari sheep breed from birth to 140 days of age. The Gompertz model was found to be the most appropriate model for male and female lambs, as it performed well under different goodness‐of‐fit criteria. However, the MMF model showed the best fit to the data for all lambs and exhibited an accuracy close to the Gompertz model for the males and females. Females had a lower AGR compared to males, and they reached their peak earlier and experienced a faster decline in AGR. The research also revealed differences in growth patterns between male and female sheep, highlighting the importance of analysing male and female data separately when describing growth patterns. As a result, as the Gompertz model was determined to be the growth curve model that best explains the growth curves of Lori‐Bakhtiari female and male lambs, it can be recommended to breeders of this sheep breed in order to determine their growth characteristics. In addition, according to the results, it can be said that it would be more correct to feed female and male lambs separately, in accordance with the possibilities of the breeders, due to AGR differences.

## AUTHOR CONTRIBUTIONS

**Saheb Foroutanifar**: Conceptualization; writing original draft; formal analysis methodology. **Majid Khaldari**: Writing–review and editing; resources; methodology.

## CONFLICT OF INTEREST STATEMENT

The authors declare no conflicts of interest.

## FUNDING INFORMATION

None.

### PEER REVIEW

The peer review history for this article is available at https://publons.com/publon/10.1002/vms3.1527.

## Data Availability

The data that support the findings of this study are available on request from the corresponding author. The authors confirm that the ethical policies of the journal, as noted on the journal's author guidelines page, have been adhered to and the appropriate ethical review committee approval has been received. The US National Research Council's guidelines for the Care and Use of Laboratory Animals were followed.
